# Mr. Bartley, on the Pernicious Effects of Quackery

**Published:** 1800-05

**Authors:** O. W. Bartley

**Affiliations:** Surgeon. Bradford, Wilts


### *To the Editors of the Medical and Phyfical Journal.*

GENTLEMEN,

IT was with pleafure I obferved in the firft volume of your very interefting and ufeful Publication, p. 337, an attack on Quackery, figned "Aliquis," and earneftly hoped the fubject would have been further profecuted, and that his juft obfervations might have excited the attention of others in their exertions to reprefs the progrefs of that growing evil, which from the daily accumulation of confequent mifchiefs, fhould become an object of the moft ferious confideration. I fhall prefume to lay before you a few remarks, which, if you confider as improper for infertion in your Journal you will fupprefs, otherwife I fhall feel myfelf gratified in their being made public.

Innumerable are the evils which arife from the blind acquiefcence of the ignorant in the fallacious pretences of itinerant pretenders, who at prefent infeft this kingdom. The numerous dupes of their defigning art are impoverifhed; population is diminifhed; the vigour and conftitutions of our countrymen are daily impaired; fcientific knowledge is defpifed; and ingenious merit goes unrewarded; whilft the unfkilful empiric fecurely lolls in the lap of luxurious eafe, and riots in oftentatious fplendour, the fruit of mifery to his fellow creatures. A circumftantial detail of the evils I mention would be painful and unneceffary, I fhall only briefly mention two or three which have fallen under my own particular obfervation.

I was acquainted a fhort time fince with a young gentleman of Briftol, who laboured fome time under a complaint, which from its fymptoms appeared to be an afcites; a regular phyfician attended him: but the diforder increafing, he grew foon impatient, and followed the advice of a perfon, who perfuaded him to take a medicine, then advertifed as a fpeedy and certain remedy for the dropfy. (The proprietor was a German, but his name, with the appellation of the medicine, I have forgotten.) Although previous to his taking this, no fymptoms of immediate danger had appeared, yet in lefs than two days afterwards, death put a final termination to the difeafe.

Another inftance I mean to adduce, is of a young man who contracted by impure connection, a fimple gonnorrhœa. Refiding with his friends, he was deterred by the dread of incurring anger, from difclofing his fituation and applying for proper medical affiftance. Having feen in a newfpaper Brodum's Botanical Syrup advertifed as a certain fpecific in this complaint, he confidently made an effay of its virtues. Buboes in the groin foon foon fucceeded its exhibition, and a confirmed fyphilis fhortly fupervened. In this deplorable fituation, the difeafe rapidly advancing, he applied to me. By charging the fyftem with mercury, and with much care and difficulty, I fubdued the virus and removed the complaint. But in confequence of the difeafe, confiderable debility, which was perhaps aggravated by the effects of the compofition he had ufed, produced atrophia, accompanied with very alarming fymptoms, (to which his youth and a conftitution extremely delicate probably contributed.) Hectic fever, œdematous fwellings in the legs and feet, great emaciation, with total lofs of appetite and ftrength; in fhort, every circumftance feemed to indicate approaching diffolution, which was however happily obviated by my advifing him a fea voyage and removal into a warmer climate, where he in courfe of time became convalefcent.

The laft I fhall now tranfcribe is of a Scotchman, who fuperintended fome work near this place on the Kennet and Avon Canal, now cutting from Bath to Newbury. From irregularity of living and exceffive drinking, he was attacked by a fevere pneumonia, which from want of attention terminated in phthifis pulmonalis. He purchafed five guineas worth of the medicine mentioned in the former cafe, whilft here in England; but not amending, he returned to Scotland, taking with him twenty-five guineas worth of that and another medicine by the fame proprietor, entitled Nervous Cordial. Notwithftanding his fecurity and dependance on thefe affiftances, death, in defiance to every opofition, foon after his arrival in his native country, put a period to his exiftence. By this account, I do not mean to infinuate that the medicine he took accelerated his death; but from it we may draw a certain conclufion, that it was not calculated to prevent it, though avowed pofitively by the proprietor.

Thefe and many other inftances may be exemplified, to fhew the pernicious tendency of encouraging fuch impofitions on the public. But what fhould excite our wonder and admiration in the greateft degree is, how a people, who pretend to rationality, fhould be fo egregioufly deluded by fuch glaring abfurdities. Can a perfon of the fmalleft penetration for a moment fuppofe, that one medicine can act as a fpecific in the cure of difeafes totally oppofite in their nature, and confequently indicating different methods of treatment in regard to regimen as well as phyfic? A moment's reflection muft fhow to him the folly of fuch a fuppofition; it fhould alfo bring to his imagination the numerous train of evils neceffarily attendant on an indifcriminate exhibition of thefe pernicious noftrums, to perfons of every defcription; no diftinction being obferved in regard to fex, fex, age, the differences in temperament and conftitution, or any other neceffary circumftance. The refult is obvious, but muft render the confideration diftreffing.

I fhall now advert to that impudent fecurity with which thefe nominal phyficians diffeminate amongft mankind, their effufions of nonfenfe, in recommendation of themfelves, the great profits accruing from their impofitions, and the avidity with which the credulous and curious multitude receive their fpurious pamphlets. A paragraph I lately obferved in one of the Briftol papers, is a fingular proof of this—it is verbatim as follows: "Of all the publications which have been ufhered into the world during the prefent century, none perhaps has met with fo rapid a fale (Paine's Rights of Man excepted) as that ineftimable production of Dr. Solomon's, entitled 'A Guide to Health.' *Forty-nine editions of* 2000, at 3s. except a few copies at Mr. Bulgin's, at Briftol, are fold; and the fiftieth of 5000 is in the prefs. This is readily accounted for, when it is confidered that there is fcarce a perfon, male or female, who are not interefted in this valuable work.—Youth and age, fingle or married, of either fex, in ficknefs or in health, fhould not lofe a moment of having it in their poffeffion. It is tranflated into the French, German, Italian and Spanifh languages, and promifes to be one of the moft valuable productions of the age.—COURIER."——This is *modeftly* introduced in the Briftol paper as a commendatory paragraph from the Courier.—Ridiculous!! This fame "*ineftimable production*," recommended here as fo *interefting* to the *fair fex*, is a compilation of obfcenity, calculated to raife in the human mind thofe very paffions it affects to affift in fubduing.—For the honour of my countrymen, I hope the above account is fallacious; if not fo, I blufh for them. It is a difhonour on our national fenfe, that fuch a jargon fhould have even paffed *two* editions.—But I intrude on your patience, I will therefore haften to a conclufion. In refpect to the means of arrefting the vaftating progrefs of this Hydra, I agree perfectly with the plan your correfpondent ALIQUIS has fubmitted to confideration; nor can I fuggeft any method I think more eligible. Without the interpofition of the legiflature it cannot however be effected. The neceffity of fuch a proceeding fhould be reprefented in ftrong terms by a committee of the firft profeffional characters in and about the metropolis, and I think there is no reafon to doubt the event would be favourable.—I have no other excufe to offer for obtruding thefe unconnected remarks on your attention, but an earneft and difinterefted defire of removing a formidable evil fo injurious to the common interefts of fociety. Relying on your indulgence, I remain, with refpect,

GENTLEMEN, your obedient fervant,

*Bradford, Wilts,*

*April* 3, 1800.

O. W. BARTLEY, Surgeon.

